# Bilateral Serous Retinal Detachment in Pregnancy

**DOI:** 10.7759/cureus.30019

**Published:** 2022-10-07

**Authors:** Daniel Sen Kai Phang, Nurulhuda Ariffin, Hayati Abd Aziz, Francesca Martina Vendargon, Khairy Shamel Sonny Teo

**Affiliations:** 1 Ophthalmology, School of Medical Sciences, Universiti Sains Malaysia, Kota Bharu, MYS; 2 Ophthalmology, Hospital Sultanah Aminah, Johor Bahru, MYS

**Keywords:** pregnancy complication, oct (optical coherence tomography), pregnancy, preeclampsia, serous retinal detachment

## Abstract

Serous retinal detachment is a rare complication of pregnancy. A 30-year-old primigravida with preeclampsia presented with bilateral blurring of vision and metamorphopsia for a one-week duration. She was referred by the Obstetrics and Gynecology department for visual assessment. Her best corrected visual acuity was 6/7.5 in both eyes. Fundus examination revealed bilateral serous retinal detachment involving maculae. She was treated conservatively and her blood pressure normalized after delivery. There was a partial resolution of subretinal fluid one-month post-delivery and a complete resolution of subretinal fluid three months later. Her final best corrected visual acuity was 6/6 and N5 in both eyes. The management of serous retinal detachment is conservative with a good visual outcome.

## Introduction

Serous retinal detachment (SRD) is a rare complication of pregnancy. The underlying pathophysiology is due to choroidal ischemia causing subretinal leakage which is a cause of serous retinal detachment [1]. The incidence of serous retinal detachment is about 1% in patients with severe preeclampsia and higher in patients with eclampsia [2]. Conservative management with optimization of the blood pressure is the mainstay of treatment. We herein report a case of bilateral serous retinal detachment in a preeclamptic lady who developed preeclampsia during her first pregnancy.

Pregnancy is known to affect all systems of the body including the visual systems. It results in metabolic, hemodynamic, and vascular changes in the ocular structures [3].

Preeclampsia is an elevated blood pressure equal to or more than 140/90 after 20 weeks of gestation with proteinuria with or without end-organ damage. Eclampsia is defined as the occurrence of convulsion in a woman with preeclampsia with no evidence of other neurological conditions. The placenta is evident as the cause of preeclampsia and eclampsia due to the failure of placenta trophoblastic invasion. Most cases of preeclampsia will resolve with the delivery of the placenta [4].

The ophthalmic manifestation of severe preeclampsia or eclampsia includes serous retinal detachment, papilloedema, central retinal vein occlusion, central retinal artery occlusion, and ischemic optic neuropathy [5]. The retinal vascular changes correlate to the severity of preeclampsia and eclampsia [6].

## Case presentation

**Figure 1:**
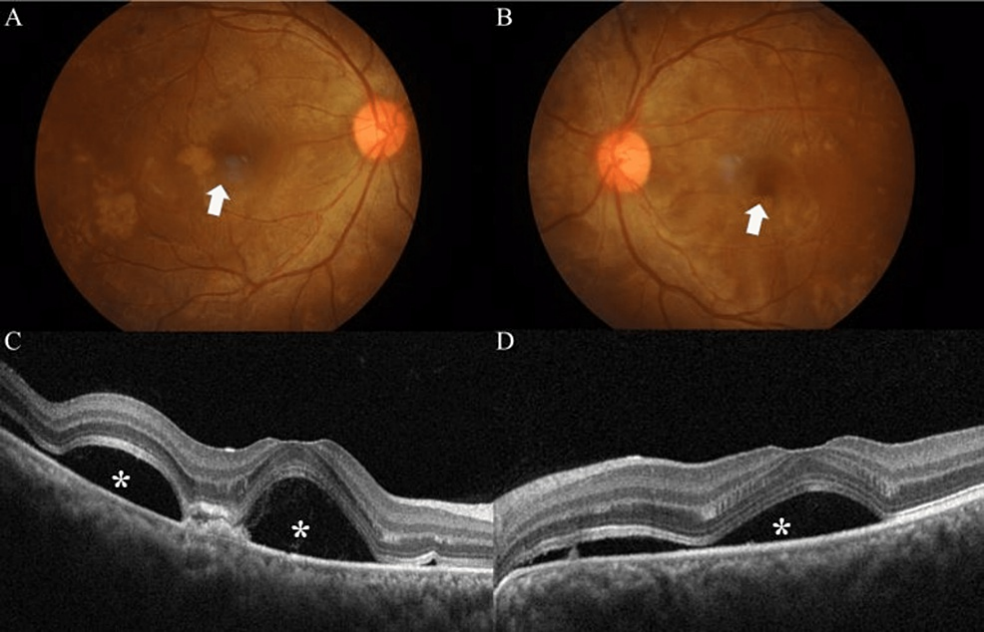
Fundus photo of the right eye (A) and left eye (B) at presentation showing bilateral serous retinal detachment involving the posterior pole (arrow). Optical coherence tomography (OCT) of the right eye (C) and left eye (D) demonstrated bilateral subretinal fluid (asterisks) more expressed in the right eye.

A 30-year-old primigravida with preeclampsia was admitted to the obstetric ward at 32 weeks of gestation for blood pressure control. Her past obstetric history revealed maternal obesity with a body mass index (BMI) of 33 kg/m2. She also had underlying gestational diabetes mellitus with a normal blood sugar profile. Her ophthalmic history was unremarkable. Examination showed a conscious lady with a blood pressure of 170/100 mmHg and the presence of bilateral pedal edema. Abdominal examination showed a singleton pregnancy at 30 weeks gestation with a normal fetal heart rate. The abdomen was non-tender and soft with normal deep tendon reflexes. Her respiratory examination did not reveal any bibasal crepitations. She was referred for eye assessment with a chief complaint of bilateral blurring of vision and metamorphopsia for a week duration since admission. Ocular examination showed the best corrected visual acuity on presentation was 6/7.5 and N6 in both eyes. Slit lamp examination of the anterior segment and intraocular pressure was normal for both eyes. However, fundoscopy showed the presence of extensive bilateral serous retinal detachment involving the posterior pole (Figure 1). There was no sign of optic disc swelling, and the retinal vessels appeared normal with no evidence of haemorrhage and cotton wool spots in both eyes.

A laboratory test was done and revealed proteinuria of 2+. Twenty-four-hour proteinuria was 9.87g/24h (standard <300mg/24 hours). Coagulation screening and renal function were within normal limits. Full blood count was normal and there was no evidence of hemolysis, elevated liver enzyme, and low platelet count (HELLP syndrome). During admission, she was started on oral labetalol 100 mg twice daily and her oral methyldopa dosage was increased from 500 mg thrice daily to 750 mg thrice daily for blood pressure control. Subsequently, her blood pressure was optimized with two oral antihypertensives and no magnesium sulphate was administered throughout her pregnancy. During her second week of admission, she developed preterm premature rupture of membrane (PPROM), and oral erythromycin 800 mg twice a day was given as prophylaxis for chorioamnionitis. She was planned for delivery via caesarean section after the completion of intramuscular dexamethasone. She delivered a baby girl with a birth weight of 1400 g with a good Apgar score.

**Figure 2:**
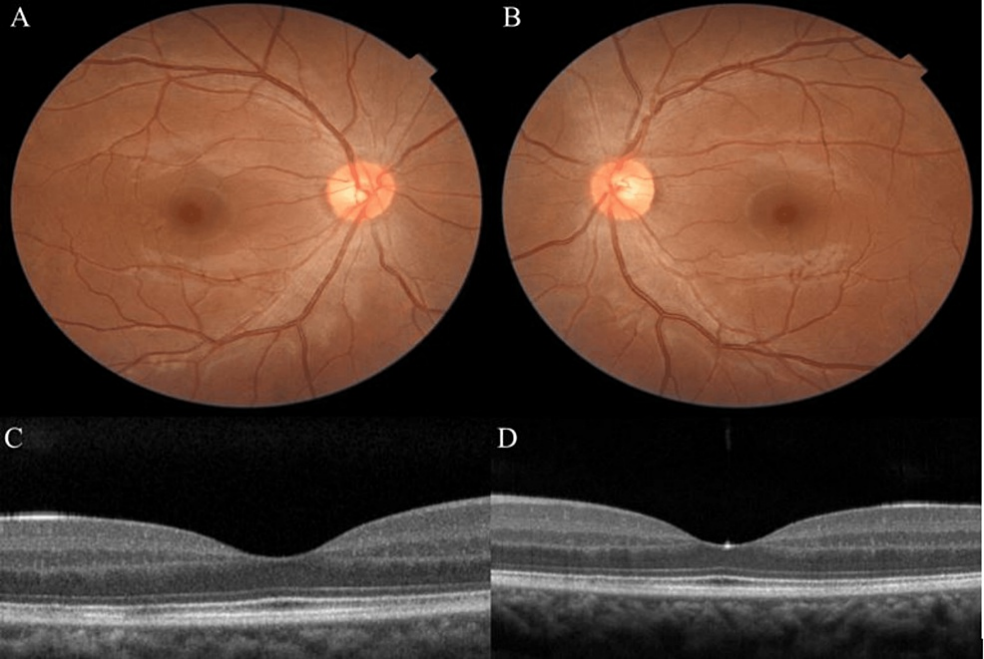
Fundus photo of the right eye (A) and left eye (B) during the follow-up period showing resolution of bilateral serous retinal detachment. Optical coherence tomography (OCT) of the right eye (C) and left eye (D) demonstrated the resolution of subretinal fluid.

She was discharged from the postnatal one week after delivery. Her metamorphopsia resolved with the best corrected visual acuity of 6/6 in both eyes. Ophthalmic examination revealed a reduction in serous retinal detachment bilaterally. During follow-up at three months, her best corrected visual acuity was 6/6 in both eyes. Fundus examination of both eyes revealed a resolution of serous retinal detachment. Optical coherence tomography of both eyes showed a complete resolution of subretinal fluid (Figure 2). The patient was followed up conservatively.

## Discussion

This case report illustrates a case of preeclampsia with bilateral serous retinal detachment. The pathophysiology of preeclampsia is not completely understood, it is associated with abnormal placentation causing increased systemic vascular resistance and vasospasm [4]. Serous retinal detachment in pregnancy is a rare complication occurring in 1-2% of pregnancies [2]. It is characterized by bilateral and serous retinal detachment. It can occur either before or after delivery [7]. 

The exact pathophysiology of serous retinal detachment in preeclampsia is unknown. However, it is thought to be due to choroidal ischemia secondary to intensive arteriolar vasospasm. Retinal pigment epithelium (RPE) receives its blood supply from choroidal circulation. Dysfunction in choroidal circulation due to ischemia can affect normal RPE functions. Subsequently, choroidal ischemia will cause degradation of the outer blood-retinal barrier and impaired fluid and ion transport function of RPE. The result is the formation of subretinal fluid which leads to serous retinal detachment [8]. The retinal vessel was not affected in our case as illustrated by the absence of retinal hemorrhages and cotton wool spots. Unlike the findings characteristically observed in central serous chorioretinopathy, optical coherence tomography (OCT) indicated there was a multiple SRD that involved areas in the macula. The ocular examination is not suggestive of Vogt-Koyanagi-Harada disease (VKH) by the absence of intraocular inflammation.

Neudorfer et al. reported that 11% of women with severe preeclampsia had abnormal fundus which includes retinal hemorrhages, macula edema, and cotton wool spots [9]. Our present case did not demonstrate any of the above findings. Her blood pressure was stabilized and reverted to normal after delivery. Komoto et al. performed a fluorescein angiography and indocyanine green in a postpartum lady with bilateral serous retinal detachment. In the early phase of fluorescein angiography, it showed a patchy delay in choroidal perfusion and in the late phase, it showed fluorescein leakage in the same area. No abnormality in retinal perfusion was detected. In the early to late phase of indocyanine green, it showed choroidal hypoperfusion [10]. Our patient refused to undergo diagnostic tests such as fluorescein angiography due to the risk to the infant during breastfeeding. Her visual acuity and metamorphopsia were significantly improved after delivery. Optical coherence tomography (OCT) is a non-invasive retinal imaging technique, that provides an instantaneous high-resolution image of the retinal layers. Unlike fluorescein angiography (FA), OCT is relatively safe in pregnant women regardless of their gestational age [9]. In our case, bilateral serous retinal detachment developed during the third trimester. An angiography study was not performed given the potential risk of teratogenicity. We performed OCT to monitor her subretinal fluid due to its advantage over fluorescein angiography.

During follow-up, the subretinal fluid in both eyes resolved gradually after delivery without needing any surgical intervention. There was also an improvement in visual acuity to 6/6 in both eyes. Our case demonstrates a similar result to the case reported by Somfai et al. who reported three preeclamptic ladies who developed serous retinal detachment of the macula after delivery in which the retinal findings were completely regressed within four to six weeks [11]. There are many cases of serous retinal detachment that have been reported in the literature [2,10,12-17]. In each of the cases, the serous retinal detachment spontaneously resolved (Table 1).

**Table 1:** Summary of reported cases of serous retinal detachment in pregnancy OU – both eyes

Reference	Age	Pre-eclampsia	Eye Involvement	SRD	Outcome
Srećković et al [12]	24	Yes	OU	Yes	Resolved
RS do Prado et al [13]	27	Yes	OU	Yes	Resolved
JTBV Raposo et al [14]	37	Yes	OU	Yes	Resolved
Asikgarip N et al [15]	29	Yes	OU	Yes	Resolved
Komoto et al [10]	31	Yes	OU	Yes	Resolved
Abdellaoui et al [16]	36	Yes	OU	Yes	Resolved
Bhandari et al [17]	23	Yes	OU	Yes	Resolved
Singh et al [2]	24	Yes	OU	Yes	Resolved
Our Case	30	Yes	OU	Yes	Resolved

## Conclusions

Serous retinal detachment in pregnancy is managed conservatively with the optimization of blood pressure. Serous retinal detachment should be suspected in a preeclamptic patient with a blurring of vision and should be referred to the ophthalmologist for a detailed visual assessment. The visual outcome is good with conservative management alone. Most patients with serous retinal detachment in preeclampsia have complete spontaneous resolution without any long-term sequelae. Nonetheless, appropriate follow-up care is mandatory in any pregnant woman with visual complaints.
